# First Observation of a Nuclear Recoil Peak at $\mathcal {O}$(100eV) with Crab: A Potential New Calibration Standard for Cryogenic Detectors

**DOI:** 10.1007/s10909-024-03252-5

**Published:** 2024-12-17

**Authors:** H. Kluck, H. Abele, J. Burkhart, F. Cappella, N. Casali, R. Cerulli, A. Chalil, A. Chebboubi, J.-P. Crocombette, G. del Castello, M. del Gallo Roccagiovine, A. Doblhammer, S. Dorer, E. Dumonteil, A. Erhart, A. Giuliani, C. Goupy, F. Gunsing, E. Jericha, M. Kaznacheeva, A. Kinast, A. Langenkämper, T. Lasserre, A. Letourneau, D. Lhuillier, O. Litaize, P. de Marcillac, S. Marnieros, R. Martin, T. Materna, E. Mazzucato, C. Nones, T. Ortmann, L. Pattavina, D. V. Poda, L. Peters, J. Rothe, N. Schermer, J. Schieck, S. Schönert, O. Serot, G. Soum-Sidikov, L. Stodolsky, R. Strauss, L. Thulliez, M. Vignati, M. Vivier, V. Wagner, A. Wex

**Affiliations:** 1 Institut für Hochenergiephysik der Österreichischen Akademie der Wissenschaften, Vienna, Austria; 2 Atominstitut, TU Wien, Vienna, Austria; 3 Sezione di Roma, INFN, Roma, Italy; 4 Sezione di Roma “Tor Vergata”, INFN, Roma, Italy; 5 Dipartimento di Fisica, Università di Roma “Tor Vergata”, Roma, Italy; 6 IRFU, CEA, Université Paris-Saclay, Gif-sur-Yvette, France; 7 CEA, DES, IRESNE, DER, Cadarache, Saint-Paul-Lez-Durance, France; 8 CEA, DES, SRMP, Université Paris-Saclay, Gif-sur-Yvette, France; 9 Dipartimento di Fisica, Sapienza Università di Roma, Roma, Italy; 10 Sezione di Roma, Istituto Nazionale di Fisica Nucleare, Roma, Italy; 11 Physik-Department, Technische Universität München, Garching, Germany; 12 CNRS/IN2P3, IJCLab, Université Paris-Saclay, Orsay, France; 13 Laboratori Nazionali del Gran Sasso, INFN, Assergi, AQ Italy; 14 Max-Planck-Institut für Physik, Munich, Germany

**Keywords:** Nuclear recoil, Neutron capture, Energy calibration, Cryogenic detector

## Abstract

Any experiment aiming to measure rare events, like Coherent Elastic neutrino-Nucleus Scattering (CE$\upnu$NS) or hypothetical Dark Matter scattering, via nuclear recoils in cryogenic detectors relies crucially on a precise detector calibration at sub-keV energies. The Crab collaboration developed a new calibration technique based on the capture of thermal neutrons inside the target crystal. Together with the Nucleus experiment, first measurements with a moderated $^{252}$Cf neutron source and a cryogenic ${{\textrm{CaWO}}_4}$ detector were taken. We observed for the first time the 112eV peak caused by the $^{182}$W(n, ${\upgamma }$)$^{183}$W capture reaction and subsequent nuclear recoils. Currently, Crab is preparing a precision measurement campaign based on a monochromatic flux of thermal neutrons from the 250-kW Triga-mark II nuclear reactor at TU Wien. In this contribution, we introduce the Crab technique, present the first measurement of the 112eV peak, report the preparations for the precision measurement campaign, and give an outlook on the impact on the field of cryogenic detectors.

## Introduction

Nuclear recoils are a versatile probe for a wide range of physics, including known processes like Coherent Elastic neutrino-Nucleus Scattering (CE$\upnu$NS) but also hypothetical processes like Dark Matter-nucleus scattering. In recent years, experiments lowered their detection threshold for nuclear recoils in cryogenic detectors down to the 10eV level, e.g., Nucleus aiming to detect CE$\upnu$NS at the Chooz power plant [1] and Cresst searching for Dark Matter [2].

At this energy scale, the calibration of the used detectors is challenging: Sub-keV radiation from a radioactive source is easily blocked before it can reach the detector or endure a significant self-absorption by the detector, which makes a homogenous, position independent irradiation unlikely. Furthermore, the energy scale of nuclear recoils is quenched compared to the scale provided by $\upbeta$ or $\upgamma$ calibration standards. As the quenching factor at these energies is poorly understood, see e.g., [3], a reliable calibration is difficult. To address these issues, the Crab (Calibrated nuclear Recoils for Accurate Bolometry) collaboration developed a new calibration standard that provides sub-keV signals and that is not quenched compared to nuclear recoils [4].

In Sect. 2 we introduce the Crab technique for nuclear recoil calibration, followed by summing up the first observation of a Crab peak in Sect. 3. We will then give an outlook on future developments in Sect. 4 before we conclude in Sect. 5.

## The Crab Technique

**Fig. 1:**
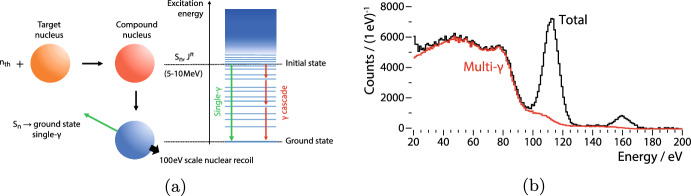
**a** The Crab technique is based on the radiative capture of *thermal neutrons* $\textrm{n}_{\textrm{th}}$ by a *target nucleus* and the subsequently *de-excitation* of the resulting *compound nucleus* to its *ground state*. In case of a *single-*$\upgamma$* transition*, the resulting *nuclear recoil* can be exactly calculated. *Multi-*$\upgamma$ *cascades* are an intrinsic background. Figure adapted from [4]. **b** The expected, total signal (*black* curve) for ${^{182}\textrm{W}}(\textrm{n},\gamma )\,\,{^{183}\textrm{W}}$ in a cryogenic ${{\textrm{CaWO}}_4}$ detector with an energy resolution of 5eV based on Geant4 and Fifrelin simulations in comparison with the multi-$\upgamma$ background (*red* curve). For details, see text

The Crab technique [4] is based on the radiative capture of thermal neutrons and the subsequent de-excitation of the created compound nucleus, as illustrated in Fig. 1: The kinetic energy of the thermal neutrons is $\approx$ 25 meV and negligible compared to the neutron binding energy $S_\textrm{n}$ of $\mathcal {O}$(10 MeV). Hence, the initial level of the compound nucleus depends only on the target nucleus. Consequently, the excitation energy is precisely known. In case the de-excitation to the ground state happens via a single-$\upgamma$ emission (*green* arrow in Fig. 1a), the decay kinematics are given by a two-body decay and the recoil energy of the de-excited nucleus can be accurately calculated. In this way, the Crab techniques overcome already two of the previously mentioned challenges for low-energy calibrations of nuclear recoil signals: Neutrons are not significantly blocked by typical detector modules, and they provide a homogeneous irradiation of the target. Also, the Crab calibration method does not suffer a quenching effect with respect to the searched for signal as both are nuclear recoils and hence directly comparable.

As we showed in [4], also the third challenge, i.e., providing a calibration signal at keV energies or below, is solvable for a wide range of common targets: Si, Ge, ${{\textrm{CaWO}}_4}$, ${\textrm{Al}_{2}\textrm{O}_3}$ all contain nuclides with a high capture cross section $\sigma _\mathrm {n,\gamma }$ for thermal neutrons, a high natural abundance $Y_\textrm{ab}$, a sizeable branching ratio for single-$\upgamma$ transition $I^\textrm{s}_\mathrm {\gamma }$, and a value for $S_\textrm{n}$ that results in nuclear recoils of $\lesssim$ 1keV.

Out of the studied nuclides, $^{182}$W has the highest figure of merit $\sigma _\mathrm {n,\gamma } \cdot Y_\textrm{ab} \cdot I^\textrm{s}_\mathrm {\gamma }$; a simulation of the expected signal is shown in Fig. 1b. To improve the precision of the simulation, the used Geant4 code [5–7] was extended by a dedicated library [8] to read-in de-excitation cascades obtain with the nuclear reaction code Fifrelin [9]. Even after applying a realistic energy resolution of 5eV, the simulation predicts a clear peak at 112eV due to the ${^{182}\textrm{W}}(\textrm{n},\gamma ){^{183}\textrm{W}}$ reaction well above the intrinsic background (*red* curve in Fig. 1b). This background is caused by all compound nuclei that de-excite via *multi*-$\upgamma$ cascades (*red* arrow in Fig. 1a). Besides the prominent 112eV peak, other peaks are expected at 160eV and 86eV, induced by neutron capture on $^{183}$W and $^{186}$W, respectively. The latter is sub-dominant to the multi-$\upgamma$ background but can be retrieved by $\upgamma$ tagging.

## First Observation of a Crab Peak

**Fig. 2:**
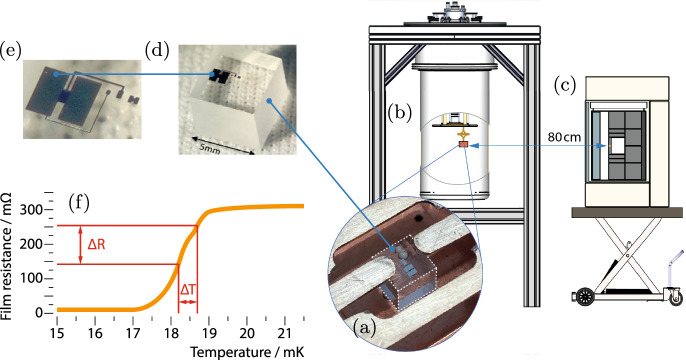
Setup used for the first observation of a Crab peak: A Nucleus detector (**a**) was placed inside a dry dilution refrigerator (Bluefors LD400, stabilized at 20 mK) (**b**) at TU Munich and irradiated with thermal neutrons. A $^{252}$Cf source ($A_\textrm{252Cf}={3.54\,\mathrm{\text {M}\text {Bq}}}$) inside a moderating box of graphite and polyethylene was placed 80 cm away from the detector (**c**), resulting in a thermal neutron rate of $\approx$ 0.25s$^{-1}$ at the detector surface. The Nucleus detector consisted of a $({5}\,\,{\textrm{mm}})^3$-cube of ${{\textrm{CaWO}}_4}$ as target with a 0.75g mass (**d**) and a Cresst-based W-thin film *Transition Edge Sensor* (TES) (**e**). As the TES is operated at the transition between the superconducting and normal conducting phase of W, small temperature changes, e.g., by nuclear recoils, result in a measurable change of the film resistance as illustrated in (**f**)

In cooperation with the Nucleus collaboration, we recently observed the predicted 112eV peak due to ${^{182}\textrm{W}}(\textrm{n},\gamma ){^{183}\textrm{W}}$ in a cryogenic ${{\textrm{CaWO}}_4}$ detector for the first time [10]; the used setup is shown in Fig. 2. We obtained a baseline resolution of 6 eV [10], well in agreement with the resolution assumed in the initial simulation (cf. Fig. 1b). Furthermore, we could reproduce the performance of Nucleus’ prototype measurement [1].

**Fig. 3:**
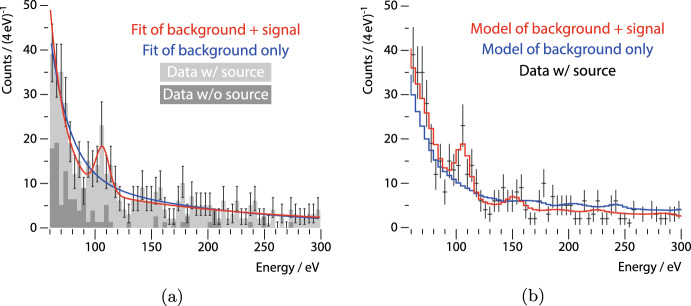
Observation of a 112eV peak due to ${^{182}\textrm{W}}(\textrm{n},\gamma ){^{183}\textrm{W}}$ in a cryogenic ${{\textrm{CaWO}}_4}$ detector: **a** Data obtained with the $^{252}$Cf source present (*light gray* histogram) show a peak which is absent in the data obtained without the source (*dark gray* histogram), the curves show a fit to the background only (*blue*) and to background plus signal (*red*); **b** comparison of the data with source (*black* data points) to an empirical model of the background (*blue* histogram) and a model of empirical background and signal as simulated with Geant4 and Fifrelin (*red* histogram). Figures adapted from [10]

The first observation of a “Crab peak” is supported by two independent approaches [10], see Fig. 3: Firstly, in data obtained with a $^{252}$Cf source present, a *peak search* favors a peak at the predicted 112eV by $3\sigma$ significance, whereas without the source no peak is observed (Fig. 3a). Secondly, a model based on the empirical observed background and the signal simulated with Geant4 and Fifrelin favors a ${^{182}\textrm{W}}(\textrm{n},\gamma )\,\,{^{183}\textrm{W}}$ signal by $6\sigma$ (Fig. 3b). Furthermore, the existence of the peak was independently confirmed by the Cresst collaboration [11]. With these observations, the feasibility of the Crab technique is proven.

## Next Steps

After we demonstrated the feasibility of the Crab method, we plan to diversify the studied targets and improve the precision of our measurements.

**Fig. 4:**
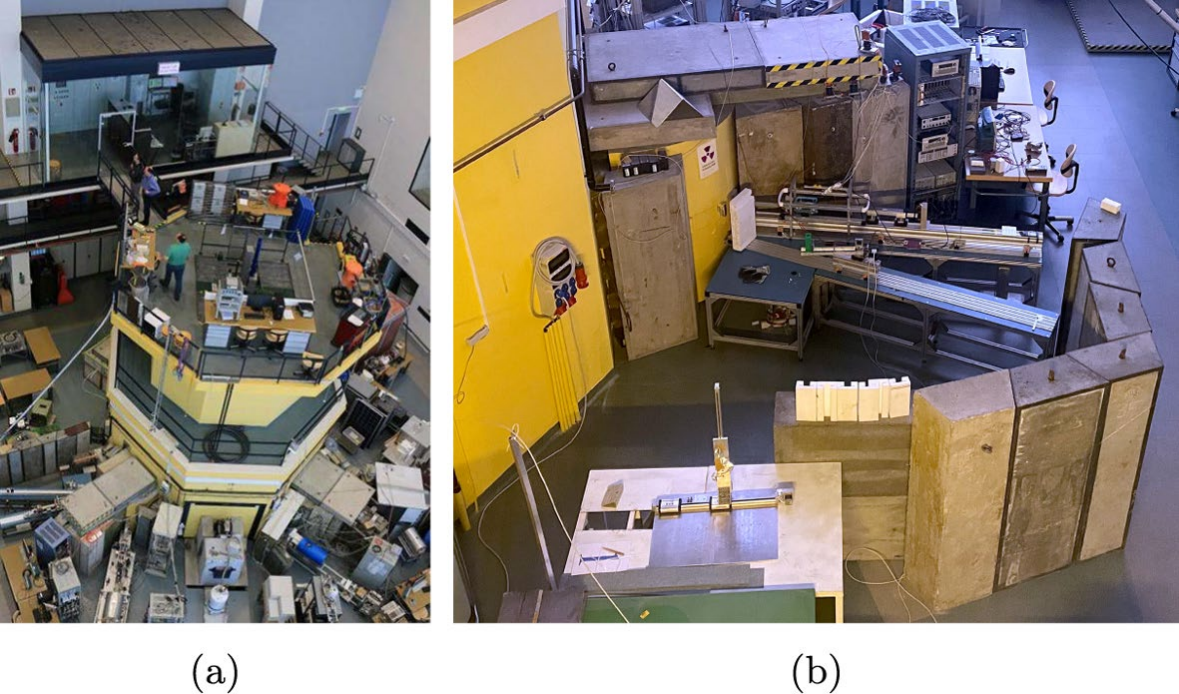
The Triga-mark II nuclear research reactor at TU Wien (**a**), where a dedicated beamline (**b**) for Crab is currently under preparation

In a second phase, we plan to increase the precision by moving the experiment to the 250-kW Triga-mark II nuclear research reactor at TU Wien, see Fig. 4. Currently, we are preparing a dedicated beam line with a monochromator, where we expect a thermal neutron flux of $\approx$ 100cm$^{-2}$s$^{-1}$. In parallel, we arrange for the installation of a “wet” $^3$He/$^4$He dilution refrigerator on site to house future Crab targets. The setup will include a Pb shield against ambient $\upgamma$ background.

Straightforward science objectives could be the measurement of the 160eV peak from ${^{183}\textrm{W}}(\textrm{n},\gamma )\,\,{^{184}\textrm{W}}$ in ${{\textrm{CaWO}}_4}$, which was not yet observed due to limited precision, as well as the 1.1 keV peak associated with $^{27}$Al in an ${\textrm{Al}_{2}\textrm{O}_{3}}$ target.

In addition, tagging of the associated $\upgamma$-rays increases the sensitivity of the Crab method and extends its application to other materials such as Ge or Si as well as quenching factor measurements. For this reason, the Crab phase 2 setup will feature a $\upgamma$ detector array around the cryostat that will consist out of 28 ${\textrm{BaF}_2}$ crystals scintillators.

**Fig. 5:**
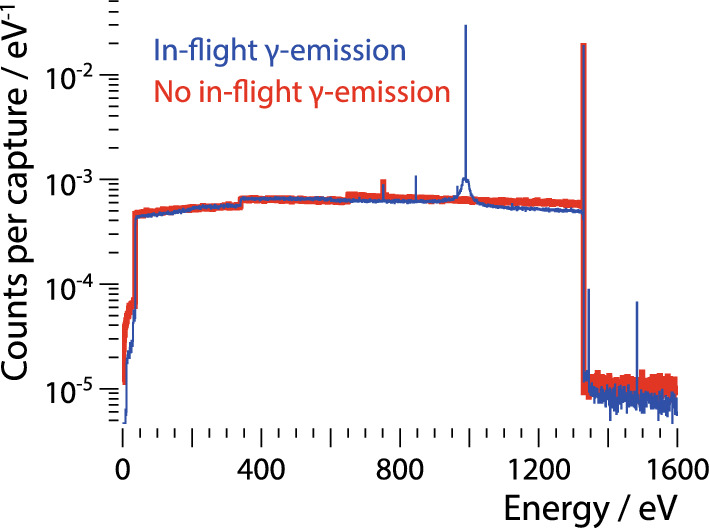
Fifrelin and Iradina simulation of the energy deposition inside a Si target induced by ${^{28}\textrm{Si}}(\textrm{n},\gamma ) \,\,{^{29}\textrm{Si}}$ under the assumption of instantaneous de-excitation (*red* curve) and de-excitation with finite timing (*blue* curve). The former shows only a prominent peak at 1330eV due to single-$\upgamma$ transition, the latter in addition a peak at 990eV due to a multi-$\upgamma$ cascade. No energy resolution is applied. Figure adapted from [12]

So far, we assumed that the de-excitation happens instantaneous. Considering the finite lifetime of intermediate states in multi-$\upgamma$ cascades in Fifrelin simulations, we found in [12] that the lifetimes of some states are with $\mathcal {O}$(100 fs) longer than the stopping process of the recoiling nucleus as simulated with Iradina [13]. Consequently, these multi-$\upgamma$ cascades can be break up in a sequence of effective single-$\upgamma$ transitions, resulting in the prediction of till now missed peaks. This allows us to apply the Crab method also to Si at sub-keV energies, as shown in Fig. 5.

## Conclusion

The Crab collaboration developed a novel, quenching-free calibration standard for $\lesssim$ 1 keV for nuclear recoil signals in cryogenic detectors based on the radiative capturing of thermal neutrons and the subsequent single-$\upgamma$ de-excitation. We successfully demonstrated its feasibility with the first observation of a Crab peak at 112eV in a ${{\textrm{CaWO}}_4}$ target. Currently, we prepare as next phase a high-precision data taking campaign at the Triga reactor at TU Wien. Furthermore, we plan to diversify the range of applicable targets by considering the timing information of multi-$\upgamma$ cascades and by applying $\upgamma$ tagging.
